# Synergistic Combination of Exogenous Hormones to Improve the Spawning and Post-spawning Survival of Female Yellow Catfish

**DOI:** 10.3389/fgene.2020.00961

**Published:** 2020-08-28

**Authors:** Weihua Hu, Peipei Huang, Yang Xiong, Wenjie Guo, Yuhong Wang, Qixue Fan, Qingyun Wang, Jie Mei

**Affiliations:** ^1^College of Fisheries, Huazhong Agricultural University, Wuhan, China; ^2^Fisheries Research Institute, Wuhan Academy of Agricultural Sciences, Wuhan, China

**Keywords:** exogenous hormones, carp pituitary extraction, spawning, post-spawning survival, female yellow catfish

## Abstract

Multiple repeat spawners make large contributions to long-term population stability and aquaculture breeding programs. A high percentage of female yellow catfish (*Pelteobagrus fulvidraco*) died for spawning failure or incomplete spawning after artificial spawning by traditional synthetic hormones including human chorionic gonadotropin (hCG), luteinizing hormone releasing hormone (LHRH), and domperidone (DOM). The present study was designed to compare the efficacy of different combinations of exogenous hormones for inducing ovulation in yellow catfish using hCG, LHRH, DOM, and carp pituitary extraction (CPE). We found a optimal strategy for exogenous hormones administration, the mixture of LHRH/CPE for the first injection and LHRH/CPE/DOM for the second injection, could greatly improve the rates of spawning success, weight of ovulated eggs and survival rate after spawning. Interestingly, a population of female yellow catfish with defective reproductive duct could not spawn and showed high mortality after induced by a combination of hCG/LHRH/DOM, whereas a synergistic combination of hCG, LHRH, DOM, and CPE could efficiently induce spawning and reduce mortality in the defective yellow catfish, in which a significant decrease of Vitellin and E2 levels. Altogether, our findings provide an effective combination of exogenous hormones to improve spawning and post-spawning survival of female yellow catfish.

## Introduction

Sexual size dimorphism has been observed in yellow catfish (*Pelteobagrus fulvidraco*), an important commercial aquaculture fish of china, in which the adult males are 2–3 times larger than the females. Commercial production of all-male yellow catfish has been successfully achieved by hormonal-induced sex reversal using sex-linked molecular markers (Wang et al., 2009; Gui and Zhu, 2012; Dan et al., 2013; Mei and Gui, 2015). With the advance of breeding technology, the production of yellow catfish is about 510 thousand tons in 2018, giving an annual increase about 15% in recent years (Zhang, 2019). However, one of the major problems is that a high percentage of female yellow catfish died after artificial spawning, because of spawning failure or incomplete spawning.

Carp pituitary extraction (CPE), the first hormone used in fish reproduction is efficient for artificial propagation of many fish species (Donaldson and Hunter, 1983). Several components of the pituitary, such as luteinizing hormone (LH, encoded by Lha and Lhb) and follicle stimulating hormone (FSH, encoded by Fsha and Fshb) were identified to regulate gonad development and maturation (Zohar and Mylonas, 2001). In addition, gonadotropin-releasing hormone (GnRH) secreted from the hypothalamus modulates the releasing of gonadotropin including LH and FSH (Herbison, 2016; Kanda, 2019). Due to the limited product of CPE and its low and unstable efficiency in some fish species (Drori et al., 1994), some synthetic agents, such as GnRH analog, human chorionic gonadotropin (hCG), luteinizing hormone releasing hormone (LHRH), and domperidone (DOM) have gradually substituted the position of CPE for artificial propagation in many fish species (Zohar et al., 1990; Peter and Yu, 1997). In catfish, either hCG (Mollah and Tan, 1983; Legendre, 1986), LHRH analog or DOM (Drori et al., 1994) has been reported to efficiently induce ovulation in females. Usually, a combination of these reagents has better effects than a single reagent.

The death of female fish after artificial spawning is usually observed in aquaculture, which is mainly caused by incomplete spawning or failure of spawning (Barry et al., 2005; Ferguson and Rice, 2006; Larsen, 2011; Teffer et al., 2017). Female sockeye salmon (*Oncorhynchus nerka*) that retained a lower proportion of eggs showed increasing reproductive longevity (Hruska et al., 2011). The gonad development and reproductive duct formation are usually affected and impaired by the environmental and genetic factors in fish species (Zupa et al., 2017). A combination of GnRH analog and DOM have been used to induce ovulation in yellow catfish (Drori et al., 1994). In yellow catfish, a high percentage of females died after artificial spawning because of spawning failure or incomplete spawning, which is harmful to the aquaculture reproduction and breeding. In addition, we also found that one large population of yellow catfish displaying defects in reproductive duct formation, could not be induced spawning by the synthetic hormones. In this study, we tried to evaluate different combinations of exogenous hormones and look for the best way to induce complete spawning and reduce the death of females after artificial spawning in yellow catfish.

## Results

### The Efficacy of Different Exogenous Hormones for Spawning Induction

To investigate the optimal exogenous hormones for artificial induction of spawning in female yellow catfish, different combinations of exogenous hormones were used (Table 1). The response of females to saline (control) and hormone treatments were summarized in Table 2, and the control females treated with saline (group 1) could not spawn. The females receiving a traditional combination of synthetic hormones efficiently spawned (group 2), but 28.33% females died 2 days after spawning. The weight of ovulated eggs in group 3 was significantly increased compared with group 2, when HCG was added in the first injection.

**TABLE 1:** The groups of yellow catfish administrated with different exogenous hormones.

		**Treatment**				
	**No. of fish**	**First injection**	**Dose (kg^–1^ b.w.)**	**Second injection**	**Dose (kg^–1^ b.w.)**	**Interval time (h)**
Group 1	15 × 3	0.7% NaCl	0	0.7% NaCl	0	12
Group 2	20 × 3	LHRH	14 μg	LHRH + HCG + DOM	16 μg + 2000IU + 10 mg	12
Group 3	20 × 3	LHRH + HCG	14 μg + 200IU	LHRH + HCG + DOM	16 μg + 2000IU + 10 mg	12
Group 4	20 × 3	LHRH + CPE	14 μg + 2 mg	LHRH + HCG + DOM	16 μg + 2000IU + 10 mg	12
Group 5	20 × 3	LHRH + CPE	14 μg + 2 mg	LHRH + CPE + DOM	16 μg + 7 mg + 10 mg	12
Group 6	20 × 3	LHRH + CPE	14 μg + 2 mg	LHRH + CPE + HCG + DOM	16 μg + 5 mg + 600IU + 10 mg	12

**TABLE 2:** The effects of different treatments on reproduction of female yellow catfish.

**Groups**	**Spawning success rate (%)**	**Latency period (h)**	**Weight of spawned eggs (g)/kg Female**	**Fertilization (%)**	**Hatching(%)**	**Mortality rate (%)**
Group 1	0^*a*^	–	0^*a*^	0^*a*^	0^*a*^	0^*a*^
Group 2	73.33 ± 3.33^*b*^	12–14	80.33 ± 5.03^*b*^	78.93 ± 4.70^*b*^	67.59 ± 5.73^*b*^	28.33 ± 3.35^*b*^
Group 3	72.00 ± 6.00^*b*^	12–14	90.33 ± 4.51^*c*^	79.11 ± 4.69^*b*^	68.98 ± 4.57^*b*^	24.67 ± 3.35^*b*^
Group 4	84.67 ± 7.02^*c*^	12–14	95.67 ± 7.63^*cd*^	82.07 ± 3.25^*b*^	69.54 ± 4.11^*b*^	20.00 ± 4.00^*c*^
Group 5	96.67 ± 3.00^*d*^	14–16	101.70 ± 6.43^*d*^	83.78 ± 1.53^*b*^	71.84 ± 4.23^*b*^	5.33 ± 1.15^*d*^
Group 6	96.00 ± 4.00^*d*^	14–16	102.70 ± 6.51^*d*^	81.35 ± 2.77^*b*^	70.77 ± 3.95^*b*^	7.33 ± 1.15^*d*^

*The mortality rate was calculated at 2 days post-spawning. Results were expressed as Mean ± SEM (standard error of mean). Means with different letter are significantly different (*P* < 0.05).*

Interestingly, after adding CPE in the first injection (group 4), the percentages of spawning success, weight of ovulated eggs and survival fish were all increased compared with group 2. When adding CPE in both the first and second injection (groups 5 and 6), a further significant increase of spawning success and survival fish was observed comparing to group 4. However, there was no significant difference between group 5 and group 6, as CPE was replaced with CPE and HCG. Our results identify an optimal combination of exogenous hormones for spawning of female yellow catfish (group 5), which could not only efficiently induce spawning of females, but also increase the rates of spawning success and survival.

### Characterization of a Population of Yellow Catfish With Defects in Reproductive Duct

**FIGURE 1:**
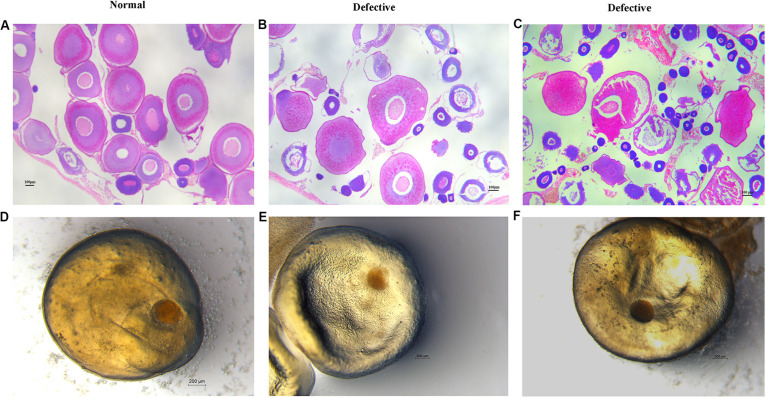
Histological examination of ovaries and oocytes in female yellow catfish. **(A–C)** H&E staining of ovaries in normal and defective yellow catfish. *N* = 3. **(D–F)** View of germinal vesicle in the oocytes of normal and defective yellow catfish. Oocytes in germinal vesicle migration stages were observed in both the ovaries of normal **(D)** and defective **(E,F)** yellow catfish. *N* = 6.

**FIGURE 2:**
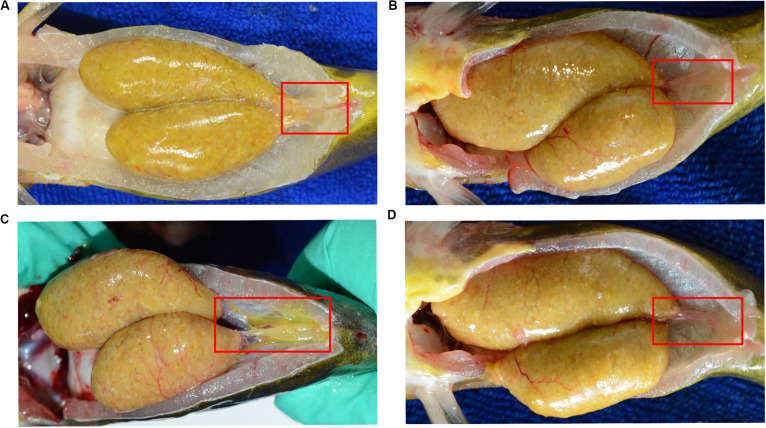
Morphological observation of reproductive duct in normal **(A)** and defective **(B–D)** yellow catfish. The reproductive duct of female yellow catfish was indicated by red square box.

Here, we found a large population of female yellow catfish (from a farming place at Honghu, city) could not spawn any eggs when induced by the synthetic hormones, the mixture of LHRH (14 μg/kg body weight) and hCG (200 IU/kg) for the first injection and the mixture of LHRH (16 μg/kg), hCG (2000 IU/kg), DOM (10 mg/kg) for the second injection (Table 3). Subsequently, we examined oocyte development and reproductive duct in these female yellow catfish. Histology analysis showed that ovaries developed normally in normal females (Figure 1A), while a number of immature and degenerated eggs were observed in the defective females (Figures 1B,C). In addition, oocytes in mature and germinal vesicle migration stages were observed in both the ovaries of normal and defective yellow catfish, suggesting that these ovaries could reach to the maturing stage (Figures 1D–F). Next, we investigated the phenotypes of oviduct in normal and defective females. Compared with the plump oviduct in normal females (Figure 2A), the defective females displayed a short and cramped oviduct (Figures 2B–D). These results suggested that the female yellow catfish (from Honghu city) could not be induced spawning by the synthetic hormones, because of their defective oviduct.

**TABLE 3:** The artificial induction of spawning in normal yellow catfish (normal) and a population of yellow catfish with defective reproductive duct (defective).

	**Treatment**						
			**Spawning**	**Weight of**			
	**First**	**Second**	**success**	**spawned eggs**	**Fertilization**	**Hatching**	**Mortality**
	**injection**	**injection**	**rate (%)**	**(g)/kg Female**	**(%)**	**(%)**	**rate (%)**
Normal	LHRH + CPE	LHRH + HCG + CPE + DOM	95.51 ± 3.37^*a*^	99.33 ± 6.81^*a*^	80.31 ± 4.71^*a*^	75.50 ± 3.55^*a*^	6.83 ± 3.50^*a*^
Defective	LHRH + HCG	LHRH + HCG + DOM	0.00^*b*^	0.00^*b*^	0.00^*b*^	0.00^*b*^	67.33 ± 10.07^*b*^
Defective	LHRH + CPE	LHRH + HCG + CPE + DOM	59.62 ± 6.04^*c*^	62.33 ± 12.06^*c*^	60.93 ± 4.07^*c*^	54.86 ± 5.81^*c*^	39.96 ± 5.81^*c*^

*The mortality rate was calculated at 2 days post-spawning. Results were expressed as Mean ± SEM (standard error of mean). Means with different letter are significantly different (*P* < 0.05).*

### A Combination of CPE and Other Synthetic Hormones Could Induce Spawning in the Females With Defects of Oviduct

In the defective females, a combination of synthetic hormones including LHRH, HCG, and DOM could not induce spawning at all, and the mortality rate is as high as 67.33% at 2 days post-spawning inducement (Table 3). Interestingly, after adding CPE in both the first and second injection, 59.62% of the treated females with defective oviduct could successfully spawn, while mortality rate after spawning was greatly reduced to 39.96%. However, all the index of reproduction in defective females were smaller than normal females, such as the rates of spawning success, weight of ovulated eggs and rates of survival. Our data indicated that a combination of CPE and other synthetic hormones could effectively induce spawning in the females with defective oviduct.

**FIGURE 3:**
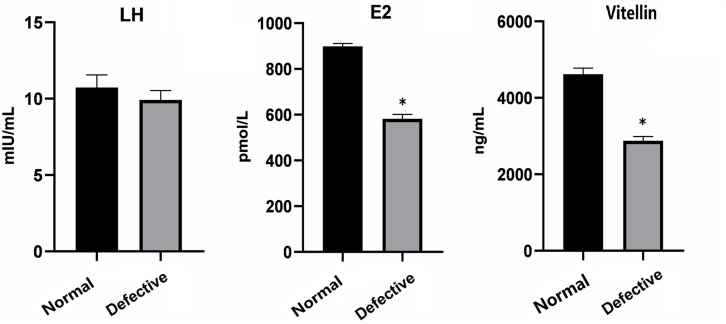
The level of LH, E2, and Vitellin in the blood of normal and defective females was examined before hormone treatment. Data expressed as mean ± SD. *P < 0.05 was considered statistically significant.

Further, we examined the levels of LH and E2 hormones and Vitellin in the blood of normal and defective females. Compared with normal, the levels of Vitellin and E2 were significantly reduced in the defective females, whereas no significant difference in the level of LH was detected (Figure 3). These data were consistent with the histology results in Figure 1, suggesting that the oocyte maturation of defective females was affected in some degree by reducing expression levels of E2 and Vitellin, while the reproductive defects may mainly due to the malformation of oviduct as the level of LH hormones did not change.

## Materials and Methods

### Experimental Fish and Hormones

Two-year-old sexually mature yellow catfish with similar size were collected from our aquaculture center at Wuhan city (Hubei province, China, normal population). And a large population of female yellow catfish at 2-year-old that showed defects in reproductive duct were collected from several ponds in the farming place at Honghu city (Hubei province, China, defective population). Each group of fish is randomly selected to conduct the experiment. All fish were acclimatized in the laboratory facility for 1 week with aerated water and under about 26°C, as previously described (Xiong et al., 2015). All experiments were carried out in accordance with the guiding principles approved by the Institutional Animal Care and Use Committee of Huazhong Agricultural University.

Four hormones or agents were used in this study. CPE is the acetone-dried pituitary extract from common carp (*Cyprinus carpio*) (Kucharczyk et al., 2008); LHRH-A2, HCG, and DOM were purchased from Ningbo Sansheng Pharmaceutical Co., Ltd. (Ningbo, China).

### Artificial Insemination and Hatching

In order to evaluate the sexual maturity, we measured body weight and gonad weight of female yellow catfish to calculate the gonad somatic index (GSI) (Srimai et al., 2019). The mean weight was 96.06 ± 7.51 g, and GSI was 13.19 ± 1.2. And the spawning stage gonads are characterized by yellow colored eggs, surface of ovaries filled with blood vessels, and ovaries occupying the entire celomic cavity.

Female yellow catfish were induced to spawn by two rounds of intraperitoneal injection with synthetic hormones, LHRH (14 μg/kg body weight) for the first injection and the mixture of LHRH (16 μg/kg), hCG (2000 IU/kg), DOM (10 mg/kg) for the second injection, a dose commonly used in the artificial propagation of yellow catfish. In addition, the male yellow catfish were injected once with a mixture of LHRH (8 μg/kg), hCG (1000 IU/kg), DOM (5 mg/kg). The sexually mature female yellow catfish from Wuhan city (normal population) were randomly divided into six groups and treated with different combinations of LHRH, hCG, DOM, and CPE. The control group was injected with 0.7% physiological saline (NaCl), and each group had three replicates (Table 1). The fish were checked every 4 h for ovulation after the second injection. The female yellow catfish (from Honghu city) with defects in reproductive duct were induced spawning by two different combinations of oxytocic hormones with or without CPE.

The semen of the male yellow catfish was mixed together for artificial insemination, with the optimal ratio of spermatozoa: egg (1.8−3.0 × 10^4^:1) according to our previous study (Zhang et al., 2017). The weight of ovulated eggs, rates of fertilization and hatching were recorded and calculated. 2 days after spawning, the survival rates of female yellow catfish in each group were determined.

### Histological Examination

For histological analysis, ovary tissues were fixed overnight in Bouin’s solution and stored in 70% ethanol. The fixed tissues were sequentially dehydrated in an ethanol series including 70, 95, and 100% ethanol. After embedded in paraffin, the ovaries were sectioned at 6 μm thickness and stained with hematoxylin and eosin (H&E) following the manufacturer’s instructions (Lin et al., 2017). The sections were examined under the microscope.

### Hormone and Vitellin Measurements

Serum were collected from 5 to 10 fish in each group and the levels of LH, E2, and Vitellin were examined using an enzyme-linked immunosorbent assay (ELISA) kit (BS-E175012, fish LH; BS-E1735702, fish E2; BS-E1740302, fish VTG; Meimian Biotechnology, Jiangsu, China) (Li et al., 2017). After measuring the optical density (OD) by spectrophotometer at a wavelength of 450 nm, the concentration of each sample was calculated from a standard curve. Each experiment was conducted in triplicate.

### Statistical Analysis

Statistical analyses were performed using SPSS 19.0 software and the results were presented as mean ± standard deviation (SD). Data in different groups were analyzed using a one-way ANOVA analysis. *P* < 0.05 was considered to be significant different.

## Discussion

Under the commercial culture conditions, ovulation obstruction and low fertility of the stripped eggs usually occur in the artificial propagation yellow catfish (Chu et al., 2014). Fish farmers have tried various synthetic hormones to promote ovulation in yellow catfish, but the outcomes are far from satisfactory in many cases. The synthetic hormones hCG, LHRH-A, and DOM have been widely used to induce fish spawning (Zohar and Mylonas, 2001). Ovulation obstruction or incomplete spawning may cause the death of female yellow catfish after induced by the traditional methods. Here we describe an efficient way to improve the rates of spawning success, weight of ovulated eggs and post-spawning survival of female yellow catfish, by a synergistic combination of exogenous hormones.

The synthetic hormones hCG, LHRH-A, and DOM have been widely used to induce spawning in catfish (Mollah and Tan, 1983; Brzuska and Adamek, 1999; Sahoo et al., 2005). GnRHa and DOM were used to induce spawning of Asian catfish, *Clarias batrachus* (Sahoo et al., 2005). The percentage of ovulating females after LHRH-A treatments was higher than the pituitary extract treatment in European catfish, *Silurus glanis* L. (Brzuska and Adamek, 1999). The rate of spawned females induced by LHRH-A was higher than by CPE in Channel catfish (Barrero et al., 2008). Therefore, CPE has been gradually replaced by the synthetic hormones to induce spawning of catfish in recent years, and a mixture of LHRH, hCG, and DOM is usually used for yellow catfish. Our study showed that a synergistic combination of CPE and other synthetic hormones could significantly improve the rates of spawning success, weight of ovulated eggs and post-spawning survival of female yellow catfish. These data suggest that CPE may contain a variety of unidentified peptide hormones that could induce fish spawning. An optimal dose of hormones are efficient and required for the induction of fish spawning, since excessive doses of exogenous hormones may cause over maturation of oocytes and have negative effects on the success rate of spawning and the quality of oocytes (Mylonas et al., 1992; Mylonas and Zohar, 2000; DiMaggio et al., 2013).

Recent studies suggest that the death of female after artificial spawning is probably caused by incomplete or failure of spawning in fish species (Barry et al., 2005; Ferguson and Rice, 2006; Larsen, 2011; Teffer et al., 2017), which is correlated with the changed levels of reproductive hormones (Larsen, 2011). Generally, GnRH secreted from hypothalamus modulates the releasing of LH and FSH in the pituitary, which regulates the secretion of sex hormones and reproductive process (Herbison, 2016; Kanda, 2019). In addition, estradiol secreted by ovarian follicles negatively regulate GnRH release (Christensen et al., 2012). Compared to the normal yellow catfish, the level of LH hormones did not change in the female yellow catfish with defects in the reproductive duct, whereas the secretion of E2 and Vitellin were significantly reduced. Vitellogenin is a precursor of Vitellin, which is the major yolk protein and the source of eggs storage (Wallace and Selman, 1990). Therefore, a number of immature and degenerated eggs were observed in the defective females (Figures 1B,C).

A synergistic combination of CPE, LHRH, and DOM could induce ovulation of defective yellow catfish, whereas the mixture of LHRH, hCG, and DOM could not. Therefore, CPE is still a good candidate for fish artificial propagation since it is rich in hormones and some factors, while the functions of some hormones have not been identified so far. Wnt4a and the miR-200 cluster on chromosome 23 are required for female reproductive duct formation in zebrafish and loss of each function led to defective reproductive duct development (Kossack et al., 2019; Xiong et al., 2020), which is similar to the phenotype of defective yellow catfish in current study. Interestingly, Oxytocin and Vasotocin are potentially responsible for zebrafish ovulation (Xiong et al., 2020). The genetic factors causing the defective reproductive duct in yellow catfish need further studies. In conclusion, we provide an optimal combination of CPE and synthetic hormones to improve the rates of spawning success, weight of ovulated eggs and post-spawning survival of female yellow catfish, which is beneficial for population stability and selective breeding program.

## Data Availability Statement

The raw data supporting the conclusions of this article will be made available by the authors, without undue reservation.

## Ethics Statement

The animal study was reviewed and approved by the Institutional Animal Care and Use Committee of Huazhong Agricultural University.

## Author Contributions

JM, WH, and PH designed the research and wrote the manuscript. WH, YX, WG, and YW performed the research. QF and QW contributed to obtain the samples. WH, JM, WG, and PH analyzed the data. All authors contributed to the article and approved the submitted version.

## Conflict of Interest

The authors declare that the research was conducted in the absence of any commercial or financial relationships that could be construed as a potential conflict of interest.
